# Two’s company, three’s a crowd: co-occurring pollinators and parasite species in *Breynia oblongifolia* (Phyllanthaceae)

**DOI:** 10.1186/s12862-018-1314-y

**Published:** 2018-12-14

**Authors:** J. T. D. Finch, S. A. Power, J. A. Welbergen, J. M. Cook

**Affiliations:** Hawkesbury Institute for the Environment, Hawkesbury Campus, Western Sydney University, Science Rd, Richmond, NSW 2753 Australia

**Keywords:** OPM, Pollination, Mutualism, *Epicephala*, *Breynia*, *Herpystis*, Host switch

## Abstract

**Background:**

Obligate pollination mutualisms (OPMs) are specialized interactions in which female pollinators transport pollen between the male and female flowers of a single plant species and then lay eggs into those same flowers. The pollinator offspring hatch and feed upon some or all of the developing ovules pollinated by their mothers. Strong trait matching between plants and their pollinators in OPMs is expected to result in reciprocal partner specificity i.e., a single pollinator species using a single plant species and vice versa*,* and strict co-speciation. These issues have been studied extensively in figs and fig wasps, but little in the more recently discovered co-diversification of *Epicephala* moths and their Phyllanthaceae hosts. OPMs involving *Epicephala* moths are believed occur in approximately 500 species of Phyllanthaceae, making it the second largest OPM group after the *Ficus* radiation (> 750 species). In this study, we used a mixture of DNA barcoding, genital morphology and behavioral observations to determine the number of *Epicephala* moth species inhabiting the fruits of *Breynia oblongifolia*, their geographic distribution, pollinating behavior and phylogenetic relationships.

**Results:**

We found that *B. oblongifolia* hosts two species of pollinator that co-occurred at all study sites, violating the assumption of reciprocal specificity. Male and female genital morphologies both differed considerably between the two moth species. In particular, females differed in the shape of their ovipositors, eggs and oviposition sites. Phylogenetic analyses indicated that the two *Epicephala* spp. on *B. oblongifolia* likely co-exist due to a host switch. In addition, we discovered that *Breynia* fruits are also often inhabited by a third moth, an undescribed species of *Herpystis*, which is a non-pollinating seed parasite.

**Conclusions:**

Our study reveals new complexity in interactions between Phyllantheae and *Epicephala* pollinators and highlights that host switching, co-speciation and non-pollinating seed parasites can shape species interactions in OPMs. Our finding that co-occurring *Epicephala* species have contrasting oviposition modes parallels other studies and suggests that such traits are important in *Epicephala* species coexistence.

**Electronic supplementary material:**

The online version of this article (10.1186/s12862-018-1314-y) contains supplementary material, which is available to authorized users.

## Background

Pollination mutualisms are amongst the most abundant and widely recognized forms of mutualism. Many different orders of insects, as well as vertebrates such as birds, bats and other mammals can act as pollinators, sometimes simultaneously [1–4]. Many plants are pollinated by several different pollinator species, whilst others are only visited by a single species [1–3]. Thus pollination mutualisms display a fascinating range of interactions from highly specialized to relatively generalized [5].

Obligate pollination mutualisms (OPMs) are specialized interactions in which female pollinators transport pollen between the male and female flowers of a single plant species and then lay eggs into those same flowers. Pollinator offspring hatch and feed upon some or all of the developing ovules pollinated by their mothers. The reward for pollination is a highly reliable food source for developing pollinator offspring. In return for this resource, pollinators provide a highly specific pollination service. OPMs have now been documented in several phylogenetically disparate plant lineages, with the best known cases involving *Ficus* [6], *Yucca* [7], Globeflowers [8] and some Phyllanthaceae [9].

Pollination mutualisms in the plant family Phyllanthaceae were first discovered in *Glochidion* [10], a genus containing approximately 300 species distributed broadly across Australia, Asia and Oceania [11]. All *Glochidion* species are believed to be involved in OPMs with *Epicephala* (Gracillariidae) moths [9]. OPMs have also been documented in several species of two additional genera within the Phyllanthaceae; *Breynia* [12] and *Phyllanthus* [13]. However, not all *Breynia* and *Phyllanthus* species have OPMs. OPMs involving *Epicephala* moths are believed to occur in approximately 500 species of Phyllanthaceae [14], making it the second largest OPM group after the *Ficus* radiation (> 750 species).

Despite the loss of plant reproductive output resulting from seed consumption, pollination by these seed predators has led plants to specialize and evolve characteristics that increased pollination efficiency by these partners. For instance, the uni-sexual flowers of *Epicephala* pollinated species from the tribe Phyllantheae have fused anthers enclosed by fused sepals [9]. These features aid in pollen collection and prevent access by less effective pollinators to pollen and nectar resources. Pollinators too, have evolved specialized morphological adaptations such as combs, pockets [15] and tentacles [16] with which to capture and manipulate pollen. In addition, many pollinators use these traits to deposit pollen deliberately through stereotyped behaviors, known as active pollination [17]. This is in contrast to the more widespread passive pollination where animals seeking nectar and pollen passively deposit pollen on flowers as a by-product of their foraging activities. The broad similarities seen between different OPMs make them useful model systems in which to study the co-evolutionary processes that give rise to and maintain mutualisms.

Strong trait matching between OPM plants and their pollinators is expected to result in reciprocal partner specificity and co-speciation [18–21] and there is strong evidence for these processes in the global radiation of *Ficus* spp*.* and their pollinating wasps [22, 23]. Strict co-speciation and reciprocal specificity, however, are not always the case in OPMs [24–30]. Recent work has shown that many *Ficus* species have more than one, and up to six, pollinator species [27, 29, 31, 32]. Multiple pollinator species are also sometimes found in *Yucca* [33] and Phyllantheae [34]. Therefore, additional processes must play a role in shaping interactions and speciation in OPMs.

Multiple pollinators on the same host plant indicate that plant and pollinator speciation are not perfectly linked. Where speciation occurs in the pollinator but not the plant, a plant will become host to co-pollinating sister species [27, 32]. Pollinators might speciate for a variety of reasons, such as in response to local climatic conditions [24, 27, 35] or by allopatric speciation [30, 33, 36]. Speciation on a single host will result in co-pollinators that are sister species and form a monophyletic group within a phylogeny. In contrast, two non-sister species may share the same host plant if one pollinator shifts from its ancestral host plant to a new one. Host shifts are thought to have occurred in several *Ficus* lineages [37–39] as well as in the Phyllantheae [30, 34, 40]. Furthermore, host shifts are seen as important processes in the evolution and diversification of herbivorous insects in general as they often precede species radiations [41–44].

Given that many OPMs involve multiple species of pollinator, an important question is “Do those species have differing effects on the fitness of their host plant?” Co-pollinators may impose different selection pressures on a shared host plant. If those pollinators have different distributions then the trajectory of co-evolution may differ between host plant populations, potentially resulting in host plant speciation [45]. Where pollinators co-occur, they may compete with each other directly. Competition between mutualists has been proposed as an explanation for secondary loss of pollination behavior, sometimes referred to as “cheating”, in some pollinator lineages [7].

In OPMs, pollinators may also occur with non-pollinating seed parasites that are unrelated to pollinators, consume seeds and do not pollinate flowers [10, 46, 47]. Like pollinators, parasites can also show high levels of reciprocal adaptation and specialization [18, 41, 48]. In OPMs, parasitic species may be as host specific as pollinators [49] or may use a broader range of host plants than co-occurring pollinators [46]. The exploitation of OPMs by parasites may reduce host plant fitness, potentially leading to the breakdown of the mutualism [19]. However, empirical evidence that parasitic species impose a significant negative impact on host species is limited [50, 51]. Identifying cheaters and parasites is important in understanding how mutualisms remain evolutionarily stable over time, as well as the relative importance of mutualism and parasitism in reciprocal specialization and co-speciation [46].

Although the broad co-evolutionary patterns of *Epicephala* and their Phyllanthaceae hosts are relatively well understood [14, 30, 40, 52], detailed knowledge of *Epicephala* diversity at the host species level is lacking. In addition, several species of Phyllantheae are known to host non-pollinating seed parasitic moths [46, 47], but their diversity and effects on the mutualism are poorly known. This is particularly true for *Breynia*, where the majority of the 70+ recognized species have not been surveyed [53]. Furthermore, there have been few wide geographic surveys of the pollinator(s) and parasites of any given Phyllanthaceae species, and almost none with dense sampling within and across sites. This will inevitably bias records towards suggesting low pollinator diversity and reciprocal partner specificity [14].

Many species of *Epicephala* have been described from Australia. However, most taxonomic descriptions do not include host plant affiliations and are based on external morphology only, which often varies little between species, making their identification difficult [54]. We investigated the number of moth species associated with the fruits of *Breynia oblongifolia,* a widespread Australian plant found along approximately 2500 km of the eastern seaboard of Australia. *B. oblongifolia* is known to host at least one species of *Epicephala* pollinator in the northern part of its range [48], but sampling has so far been limited to a single site. If multiple *Epicephala* species are found, then do they have distinct geographic distributions? Do all the *Epicephala* species actively pollinate *B. oblongifolia* or are non-pollinating “cheater” species also present? What is the phylogenetic relationship of any co-existing species? And finally, what other species, if any, use the fruits of *B. oblongifolia*? We used a mixture of DNA barcoding, genital morphology and behavioral observations to determine the number of moth species in the fruits of *Breynia oblongifolia*, their geographic distribution, pollinating behavior and phylogenetic relationships. Our aim is not to provide species descriptions but to use the aforementioned techniques to test the assumption of strict reciprocal specificity in *B. oblongifolia*. We hope that this study will help to inform future taxonomic work as well as furthering understanding of the diversity, ecology and evolutionary history of pollinators involved in OPMs.

## Methods

### Sampling methods

We sampled *Breynia oblongifolia* fruits once from each of six study sites (Table 1) over approximately 750 km along the east coast of New South Wales (NSW), Australia. Two to six fruits were picked haphazardly from each of 15–30 plants per sampling site, depending on the number of plants and mature fruits. With the exception of Richmond, all sites were coastal and in close proximity (< 200 m) to the ocean shoreline. Fruits were only sampled when larger than 5 mm in diameter and beginning to redden, indicating fruit maturity. Fruits that already had emergence holes were avoided. Fruiting time varied between sites, so it was not possible to collect fruits from all sites at the same time of year.

**Table 1:** Fruit, plant and insect sampling by site

Site	Location	Plants	Fruits	Date	Insects Reared	Insects Caught	Shared
Copacabana (CC)	−33.492065, 151.429909	28	82	Jan-16	39	0	4
Narooma (NR)	− 36.216884, 150.136214	15	90	Jan-17	20	0	0
Richmond (RC)	−33.620612, 150.738481	30	90	Dec-15	30	77	0
Shoalbay (SB)	−32.717359, 152.166252	36	61	Apr-16	25	9	2
Shellharbour (SH)	−34.595138, 150.897278	30	85	Jan-16	16	0	0
Sawtell (SW)	−30.380541, 153.090797	22	90	Jan-16	23	0	2

Insects reared is the number of adult insects reared from collected fruit. Insects caught is the number of wild caught adult insects captured following oviposition. Shared is the number of plants per site that were found to host both *Epicephala* species

Fruits were placed singly in 70 ml plastic pots fitted with nylon mesh covered holes to allow for air circulation. The pots were stored at room temperature to allow insects to emerge. Insects emerged as larvae before pupating in the tubes. Adult insects were killed by freezing and then stored in 95% ethanol prior to DNA extraction. Fruits were dissected after 2 months to check for insects that had died or failed to emerge.

### DNA barcoding

For DNA extraction, two whole legs were removed from each adult insect, dried and placed in 100 μl of a 5% Chelex® 100 Resin - TE solution (1 M Tris pH 8.0, 0.5 M EDTA) [55] in 1.5 ml centrifuge tube. The sample tissue was homogenized using a 5 mm stainless steel ball bearing in a Tissuelyser II (Qiagen, Venlo) at 30 Hz for 2 min. Cell disruption was performed by heating to 96 °C for 15 min. The solution was then centrifuged at 13,000 rpm for 15 min. The resulting supernatant was removed by pipet and stored at − 20 °C.

DNA barcoding was used to delimit insect species, by amplifying and sequencing a fragment of the mitochondrial cytochrome c oxidase subunit I (COI) gene using the lepF1 and R1 primers [56]. PCR reactions were as follows: 1.2 μl of 10x NH4 Reaction Buffer (Bioline, Australia), 0.5 μl of 25 mM MgCl2, 2.5 μl of 25 mM dNTP in Tris-HCL [pH 8.0], 4 μl of ~ 1 ng/ μl genomic DNA in TE, 0.5 μl of 10 pmol forward and reverse primer, 0.5 μl of BIOTAQ™ (Bioline, Australia) and made up to 12 μl total volume with Ultrapure Water (Invitrogen, California). PCR reactions were performed on a DNA Engine Dyad Peltier Thermal Cycler (Bio-Rad, NSW). The cycling conditions were 95 °C for 5 min followed by 35 cycles of 94 °C for 40 s, 47 °C for 60 s and 72 °C for 70 s and a final elongation period of 72 °C for 5 min. PCR amplification was checked by running aliquots of the reactions on a 1.5% TBE-agarose gel stained with SYBR™ Safe DNA Gel Stain (ThermoFisher Scientific, NSW) and visualized under UV light. Amplified DNA fragments were sequenced unidirectionally at the Hawkesbury Institute for the Environment on an Applied Biosystems genetic analyzer 3500 (Applied Biosystems, California) using BigDye Terminator kit (v3.1, Applied Biosystems, California). Sequencing reactions were performed with 5 pmol of the forward primer and ~ 20 ng of DNA in 10 μl of Ultrapure Water (Invitrogen, California).

Sequences were then grouped into *Epicephala* and non-*Epicephala* insects. COI sequences from our *Epicephala* moths were aligned with the reference sequence, *Epicephala* sp. *Breynia oblongifolia*, (GenBank: FJ235381.1) using the Geneious alignment tool in the Geneious® Program (10.3.3). Any nucleotides with more than a 5% probability of error were removed from the ends of sequences and sequences < 300 bp long were discarded from the analysis. Sequence divergences were displayed as Neighbor Joining (NJ) Consensus Trees in Geneious® using the Tamura-Nei genetic distance model with 200 bootstrap replicates. The output of the NJ trees was used to calculate the number of species per sampling site and visualized using ggplot2 [57] and rgdal Library [58] in RStudio (Version 1.0.153) [59].

Alignments of *Epicephala* sequences from Geneious® were exported into Rstudio (R Version 2.14.0) [59] and analyzed using the Spider [60] and Ape [61] packages to generate a pairwise distance matrix and calculate the inter and intra species pairwise genetic distances. The sequence from the *Epicephala* moth previously sampled from *Breynia oblongifolia* by Kawakita et al., [14] nested within our species B and as such was included in the species when calculating the inter and intra species pairwise percentage distances.

COI sequences from non*-Epicephala* moths were identified using BLAST searches in the Geneious® program. Where the percentage pairwise identity between the query sequence and the hit with the greatest percentage pairwise identity was greater than 99%, we assumed that the two sequences belonged to the same species. All queried sequences belonged to an undescribed species of Tortricidae. To determine if this is closely related to totricids found feeding on seeds in other Phyllantheae fruits [46], we aligned sequences from that study with our own using ClustalW alignment tool in the Geneious® program and calculated their pairwise percentage similarity. COI sequences from non-*Epicephala* moths were deposited in Genbank® (Accession numbers: MH544592-MH544609).

### Phylogenetic analysis

We conducted a phylogenetic analysis to determine the evolutionary relationships of the *Epicephala* species on *B. oblongifolia*. In addition to COI, we amplified and sequenced sub-units of the nuclear genes, elongation factor 1-alpha (EF-1α) and arginine kinase (ArgK) [62], for five individuals from each of the *Epicephala* species identified by the NJ tree, using individuals from the Richmond and Narooma sampling sites. The PCR conditions were as for the COI gene fragment but annealing temperatures were 58 °C for both the EF-1α and ArgK primer pairs. Sequencing reactions were the same as COI but we used the reverse primer for EF-1α as this gave higher quality results. The COI, EF-1α and ArgK nucleotide sequences used for the phylogenetic analysis were deposited in GenBank® under the accession numbers MH480583-MH480602 (COI and ArgK) and MH544582-MH544591 (EF-1α.) Additional sequences for *Epicephala* individuals from *Breynia* spp. sampled and sequenced from previous studies [9] were obtained from GenBank®, accession numbers: COI (FJ235373.1-FJ235391.1, PopSet: 2195523650), EF-1α genes (FJ235491.1-FJ235514.1, PopSet: 219552572) and ArgK (FJ235392.1-FJ235415.1, PopSet: 219552403). One *Epicephala* species, *Epicephala* sp. ex *Phyllanthus koniamboensis*, and three non-*Epicephala* Gracillariids were used as outgroups: *Cuphodes diospyrosell, Melanocercops ficuvorella* and *Stomphastis labyrinthica*.

Alignments were performed separately for each gene with the ClustalW tool in the Geneious® Program. The COI (429 bp), ArgK (533 bp) and EF-1α (436 bp) alignments were then concatenated into a single 1398 bp sequence for each individual for phylogenetic analysis. We used PartitionFinder2 [63] to identify the most suitable partitioning scheme and model of molecular evolution for each partition based on AICc scores, specifying linked branch lengths. The best partitioning scheme identified by PartitionFinder2 had 8 partitions and used 6 molecular evolution models. However, this partitioning scheme frequently suffered from numeric instability and low ESS scores (< 20). As such we opted to use an intuitive partitioning scheme separating the nuclear and mitochondrial genes into two groups and modelling the 1st and 2nd codon positions separately from the 3rd codon position in each group [63–66]. The concatenated alignment was exported to Beauti2 and we conducted our analysis in BEAST v2.4.7 [67]. For both models, we specified the most parameter rich yet least restrictive model, GTR + I + G [68] as the substitution model for all 4 partitions. We used a strict clock model with clock rate set to 1 and specified a Yule Tree model with the chain length set to 10,000,000, a burn in of 10% and sampled every 1000 iterations. The models were run in BEAST with a random number seed and the outputs of the models was visualized with Tracer v1.6.0 [69].

### Species delimitation and hypothesis testing

Our analysis indicated that the two *Epicephala* species on *B. oblongifolia* are distinct species. However, the relationships between these species and the *Epicephala* collected from other *Breynia* host plants remained unclear. As such, we used Generalized Mixed Yule Coalescent models (GMYC) [70] in the R package Splits [71] and Poisson Tree Processes (PTP) models [72] to perform species delimitation using the maximum clade credibility tree generated by BEAST. For the GYMC analysis we specified a “single-threshold” and otherwise used the default settings. For the PTP analysis we specified a chain length of 100,000 and a burn in of 10%.

We tested the alternative hypothesis that the two *Epicephala* species identified by this study form a monophyletic group (i.e. are sister taxa) in BEAST. To do this we then created an alternative model in which we constrained the phylogenetic tree topology to force all our *Epicephala* sequences from *Breynia oblongifolia* to form a monophyletic group. We then used the Path Sampler application in BEAST to estimate marginal likelihoods for both models [73]. We ran two separate analyses of 8 and 50 steps, both using a chain length of 1,000,000, an α value of 0.3 and a burn in of 50%. We compared the two models by calculating the corresponding Bayes Factors, defined as the difference in the log marginal likelihoods between the two models [74].

### Genital dissections

Male and female genitalic morphology can also be useful in the identification of *Epicephala* species, as the reproductive structures in both sexes show a high degree of morphological variation [54]. We dissected the genitalia of a subset of 10 males and 10 females from the two species identified from molecular data, in order to determine if morphological differences also existed between them. Dissections and slide preparation were performed under a Leica MZ FLIII stereomicroscope (Leica Microsystems, Wetzlar, Germany). Abdomens were removed from adult *Epicephala*, placed in a 10% KOH solution and heated to 100 °C before being allowed to cool for 5 min. For imaging, genitalia were stained using a 1% Chlorazol Black solution (Sigma-Aldrich, Missouri, USA) and dehydrated in a series of 70–100% ethanol solutions before being mounted in Euperal (ASCO Laboratories, Manchester, England) on a glass slide. Imaging of the genitalia was performed using a Leica DCF 500 camera fitted to a Leica M205A stereomicroscope (Leica Microsystems, Wetzlar, Germany). We took images at multiple depths and stacked them using Zerene Stacker (Zerene Systems, Richland, USA). After noting considerable differences in the females’ ovipositors we also dissected and imaged the ovaries of both species. We dissected the abdomens of 6 females from both species collected at the Richmond site in a 30% ethanol solution and imaged them in solution as detailed above.

### Pollination observations

We observed *Epicephala* moths pollinating and ovipositing into *Breynia oblongifolia* flowers at the Richmond site (RC) from 13/09/2017 to 22/04/2018 on up to four evenings per week. We also made attempts to observe pollination behaviors at the Shoal Bay site (SB) on the evening of 15/01/2017. At RC and SB, *B. oblongifolia* makes up a large proportion of the woodland understory and occurs there at all growth stages. Pollination behaviors were observed shortly after sunset when the moths became active. White LED headlights were frequently found to disturb pollinating moths, so moths were located and observed using red LED headlights, which did not noticeably alter their behavior. When a moth had been observed to pollinate and oviposit into a flower, the moth and flower were removed and taken back to the lab for identification and further study.

The collected flowers were dissected under a EZ4 W Stereo Microscope (Leica Microsystems, Wetzlar, Germany) and the number of pollen grains and location of oviposited eggs in the flowers were determined. Moths were killed by freezing, identified by genital dissection and checked for pollen under a stereo microscope. We used a Welch’s t-test to determine if the number of pollen grains deposited by females differed between species. Images of females of both species with pollen were obtained using a Phenom XL Scanning Electron Microscope (Phenom World, Eindhoven, Netherlands) at the Advanced Materials Characterization Facility at Western Sydney University, Parramatta. Data on pollination observations were deposited in figshare [75].

## Results

### DNA barcoding

COI sequences > 300 bp were obtained for 134 *Epicephala* spp*.* moths from the six sampling sites (Table 1). Neighbor joining trees of COI pairwise distances revealed two species (A and B) associated with *Breynia oblongifolia* (Additional file 1)*.* The sequence of the *Epicephala* moth previously sampled from *Breynia oblongifolia* by Kawakita et al. [14] nested within our species B.

**Fig. 1:**
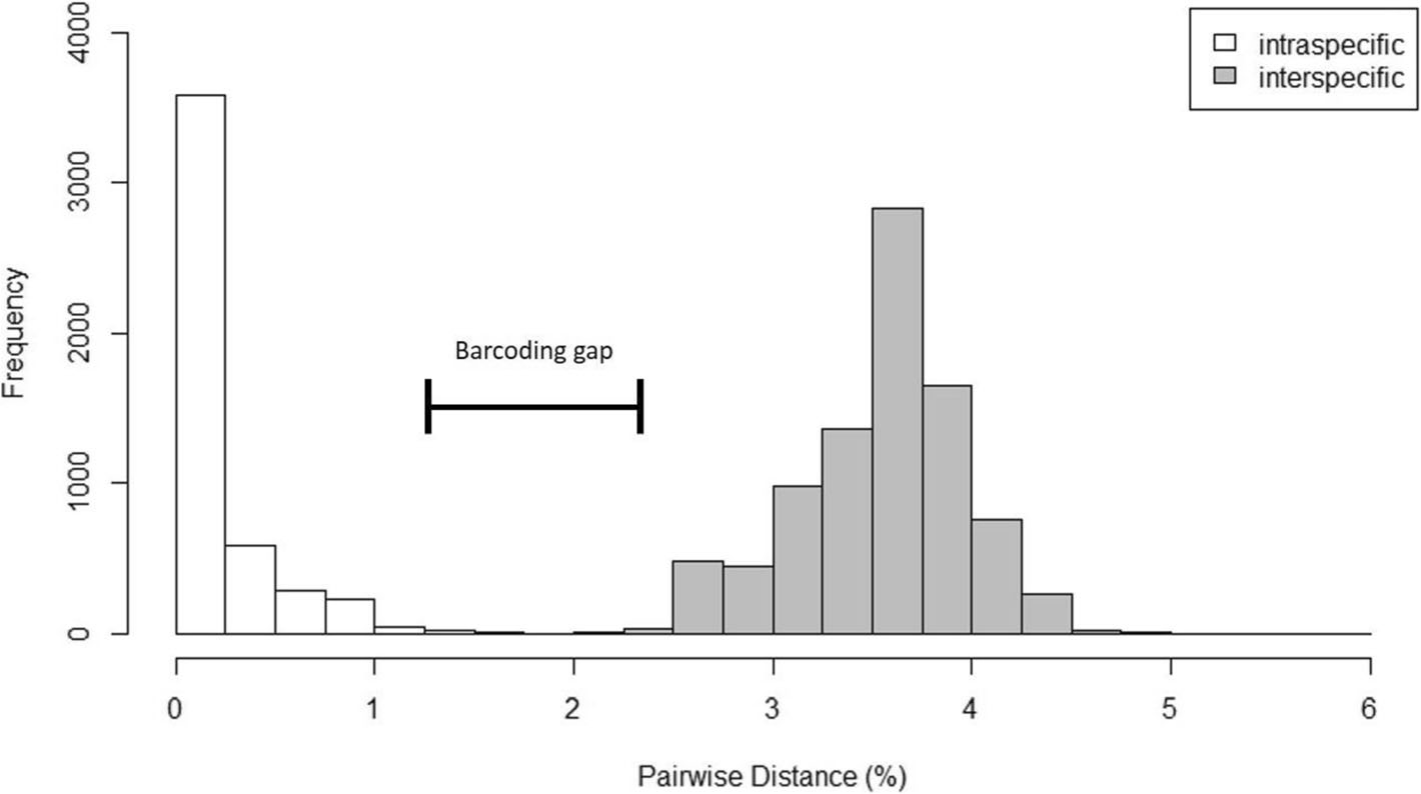
Frequency distribution of intraspecific and interspecific pairwise percentage distances in a sub-unit of the *COI* gene sequenced from *Epicephala* species **a** and **b**

The mean pairwise genetic distance within *Epicephala* species was 0.37 and 0.08% for A and B respectively. In contrast, the mean pairwise distance between individuals of the different species was 3.54%. The distribution of pairwise percentage distances between species (interspecific) greatly exceeded and did not overlap with pairwise distances within species (intraspecific), resulting in a “Barcoding Gap” [76] (Fig. 1). We consider that these results support the existence of two *Epicephala* species on *B. oblongifolia*.

**Fig. 2:**
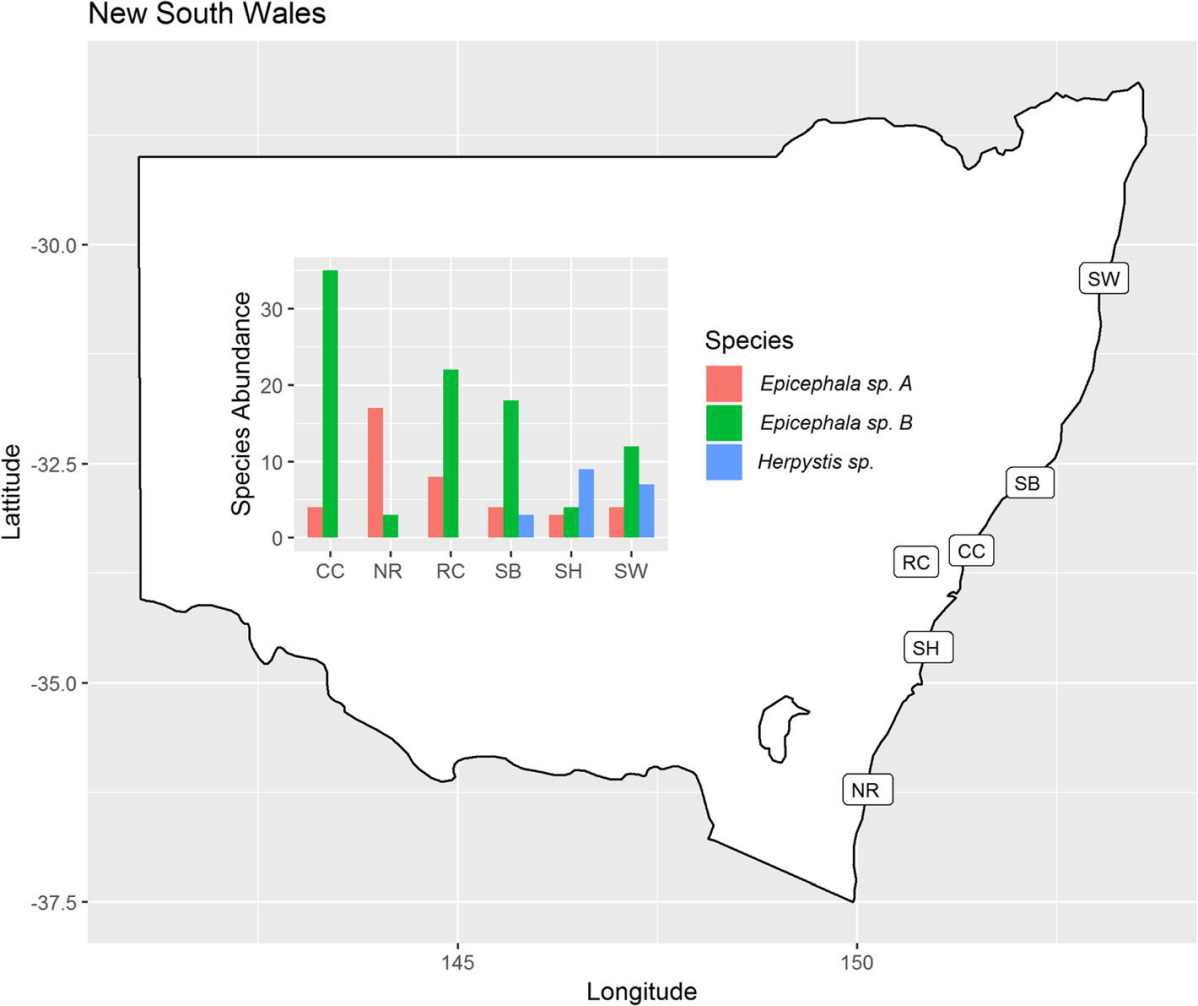
Distribution of sampling sites in New South Wales and relative abundance of *Epicephala* and *Herpystis* species collected from *Breynia oblongifolia* as determined by Neighbor Joining Consensus Trees of COI mitochondrial sub-units

Both *Epicephala* spp. A and B were found at all sites (Fig. 2), with no obvious differences in geographic distribution. At a finer scale, *Epicephala* spp. A and B were collected from fruits on the same individual plant at three sites on eight different plants (Table 1). The relative abundance of the species differed between sampling sites with B dominant at most sites but A most abundant at Narooma (NR), the most southerly sampling site.

Moths other than *Epicephala* (*n* = 19) were found to inhabit *B. oblongifolia* at 3/6 sites (Fig. 2). The mean pairwise genetic identity for those moths was 99.37% (sd = 0.44), suggesting a single species. In addition, this species was found to have a > 99% pairwise identity to *Herpystis* sp. ANIC1 (Tortricidae)(museum voucher: 11ANIC-12,766, BOLD: AAX1579). *Herpystis* sp. ANIC1 is an as yet undescribed specimen in the Australian National Insect Collection (CSIRO, Black Mountain, Canberra), and was sequenced as part of the International Barcode of Life Project [77]. This suggests strongly that our moths belong to *Herpystis* sp. ANIC1 and they will henceforth be referred to as such. When we compared the COI sequences of *Herpystis* sp. ANIC1 and *Tritopterna* sp. AK-2010, another torticid moth taken from the fruits of *Glochidion* (Phyllantheae), the pairwise identity was 87.6% (sd = 0.50). We attempted to create a phylogeny for *Herpystis* sp. ANIC1 but found no sequences were available in any public database for any other member of this genus.

### Phylogenetic analysis

**Fig. 3:**
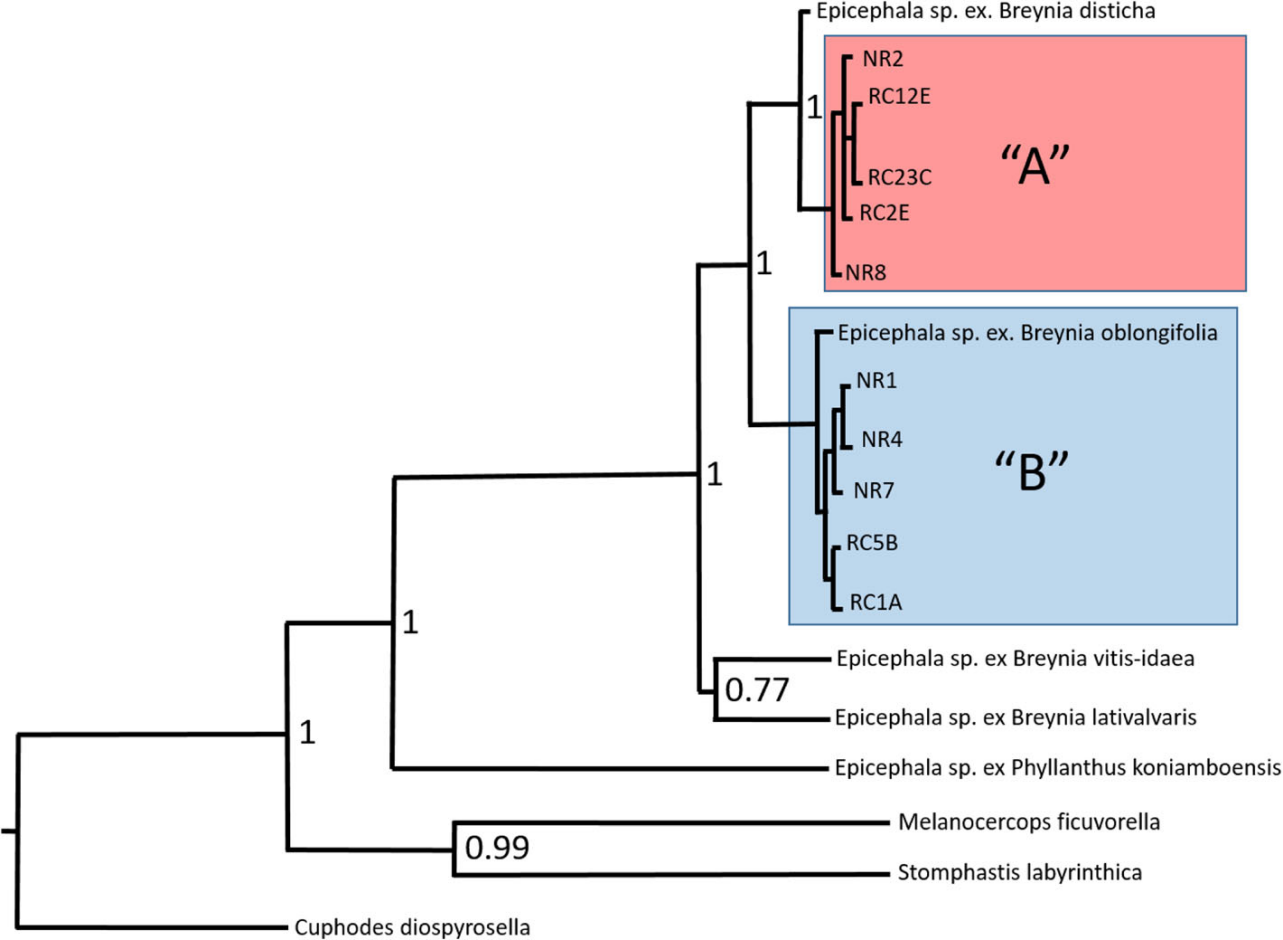
Bayesian phylogeny of *Epicephala* spp. collected from *Breynia* spp. generated by BEAST using concatenated COI, EF-1α and ArgK nucleotide sequences (1389 bp). Species names were included where known, undescribed species were labeled by host plant. Posterior support values are given adjacent to selected nodes. *C. diospyrosella*, *M. ficurvorella*, *S. labyrinthica* and P. *koniamboensis* were used as outgroups. Sample names refer to sampling location; Narooma (NR) and Richmond (RC)

Both species of *Epicephala* from *B. oblongifolia* grouped within the *Epicephala* moths sampled from other *Breynia* species. The *Epicephala* collected from *B. oblongifolia* were found not to be monophyletic. Species A was most similar to, but distinct from one *Epicephala* moth collected from *Breynia disticha* in New Caledonia (Fig. 3). Percentage similarity in the COI gene between that insect and our own was 97.9–98.6%, whilst within species A, percentage similarity was slightly higher at 98.8–99%. As such, it is not clear if the insects from *B. disticha* is a distinct species but it is certainly closely related to our species A. The lack of other sequences for moths from *B. disticha* prevents further analysis for now. The *Epicephala* moth previously collected from *Breynia oblongifolia* [14] was found to be closely related to our species B. The percentage similarity in COI sequences between *Epicephala* sp. ex. *B. oblongifolia* and our own was between 98.6–99.7%. Percentage similarity within clade B was comparable at 98.7–99.2%, indicating that *Epicephala* sp*.* ex*. B. oblongifolia* and our clade B likely belong to the same species (Fig. 3). Overall the phylogenetic analysis indicates that *Breynia oblongifolia* hosts at least two species of *Epicephala*.

### Species delimitation

The GYMC analysis determined that our *Epicephala* species A and B are separate species and that the *Epicephala* moths previously collected from *B. oblongifolia* [14] were the same species as our species B. Furthermore, it also separated our species A and the *Epicephala* previously collected from *B. disticha* in New Caledonia into separate sister species. However, yule support values for nodes in the GMYC analysis were low (< 0.45). In particular, support values for the separation of species A and *Epicephala* sp. ex. *B. disticha* was very low at 0.2. Conversely, although the PTP analysis agreed that species A and B were distinct and that *Epicephala* sp. ex. *B. oblongifolia* belonged to our species B, that analysis grouped species A and *Epicephala* sp. ex. *B. disticha* into a single species that occurs in both Australia and New Caledonia. Again, the lack of multiple sequences for *Epicephala* sp. ex. *B. disticha* limits our ability to resolve this issue.

### Species hypothesis testing

For the 8 step run, the marginal log likelihood of the unconstrained tree was − 5148.63 compared with − 5225.65 for the constrained monophyletic tree. For the 50 step run, the marginal log likelihood of the unconstrained tree was − 5183.81 compared with − 5293.23 for the constrained monophyletic tree. The log Bayes Factors for the 8 and 50 step runs were 104.02 and 109.47 respectively which strongly suggests that species A and B do not form a monophyletic clade [74, 78].

### Genital dissections

**Fig. 4:**
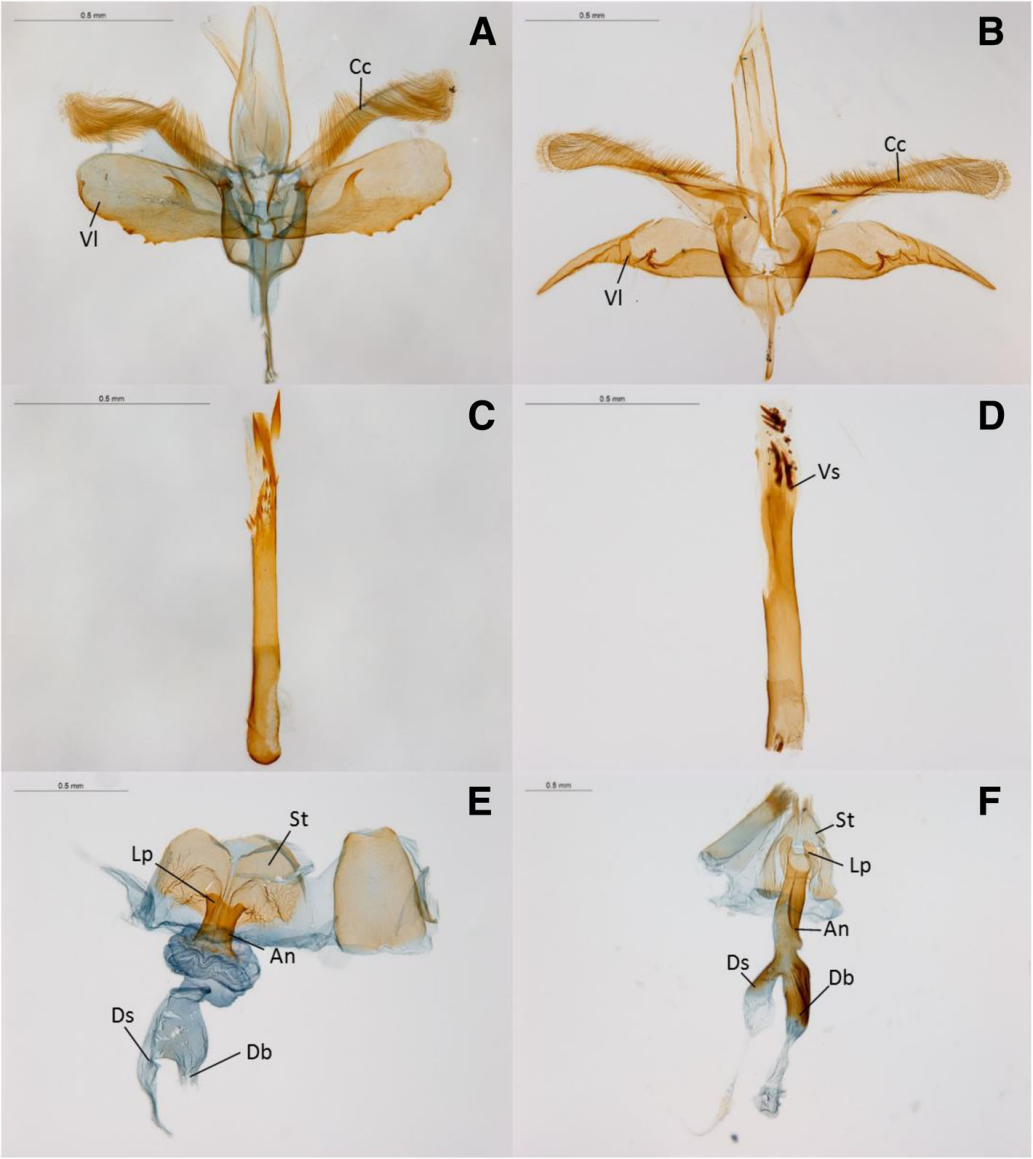
Reproductive structures of *Epicephala* moths collected from *Breynia oblongifolia*. Left side, species (**b**), right side, species (**a**). Males: **a** and **b** Male genitalia, **c** and **d** Phallus. Females: **e** and **f** Female genitalia. Labels refer to valvae (Vl), cucullus (Cl), vesicle (Vs), sternite (St), lamella postvaginalis, antrum (An), ductus bursae and ductus seminalis (Ds)

In both sexes, genital morphology differed between species. Males of species B possessed a varying number of irregular “teeth-like” structures along the ventral edge of their obtuse valvae, as well as a pair of posteriorly pointed hooked spines along their inner surface (Fig. 4a). The valvae of males from species A were falcate in shape. In addition, the ventral edge of base of the valvae showed a strong sigmoidal curve which terminated in an acute spine pointed toward the phallus (Fig. 4b). The cucullus of species A was straight with a hairy inner surface and a rounded distal end, whilst in species B the cucullus was flat ended, obtusely angled ventrally and was more densely haired than species A. The phallus of males from species B possessed a variable number of large posteriorly orientated sclerotized spines which were largest apically (Fig. 4c). Conversely, males from species A showed a single small sclerotized longitudinal ridge with a small obtuse spine on the outer surface at the posterior end (Fig. 4d). In addition, several sclerotized spines were visible on the vesicle of males from species A.

**Fig. 5:**
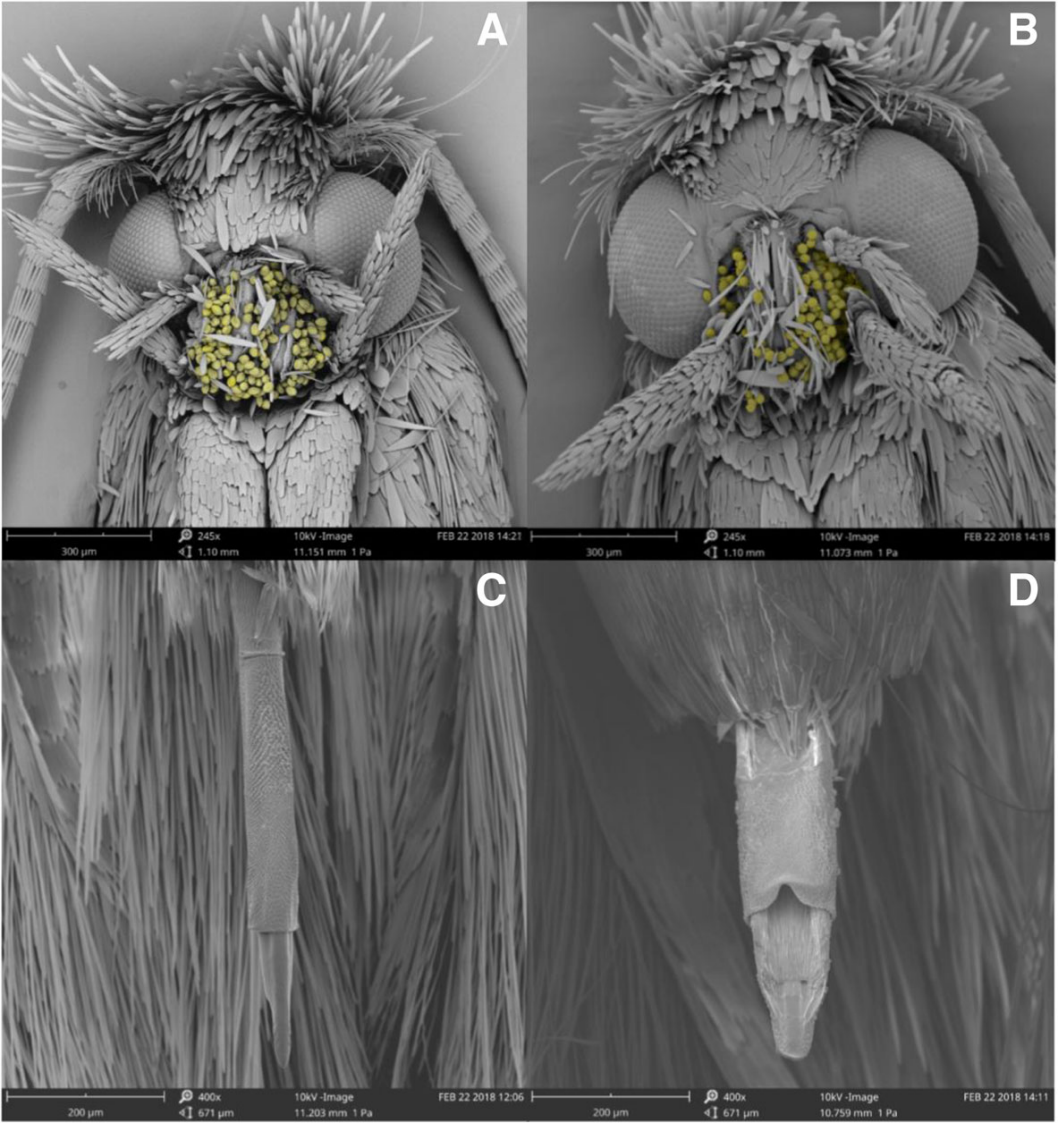
Scanning electron microscope (SEM) images of *Epicephala* moths collected from *Breynia oblongifolia*. Left side, species (**a**), right side, species (**b**). **a** and **b** Females with pollen artificially coloured yellow, **c** and **d** ventral view of female ovipositors

**Fig. 6:**
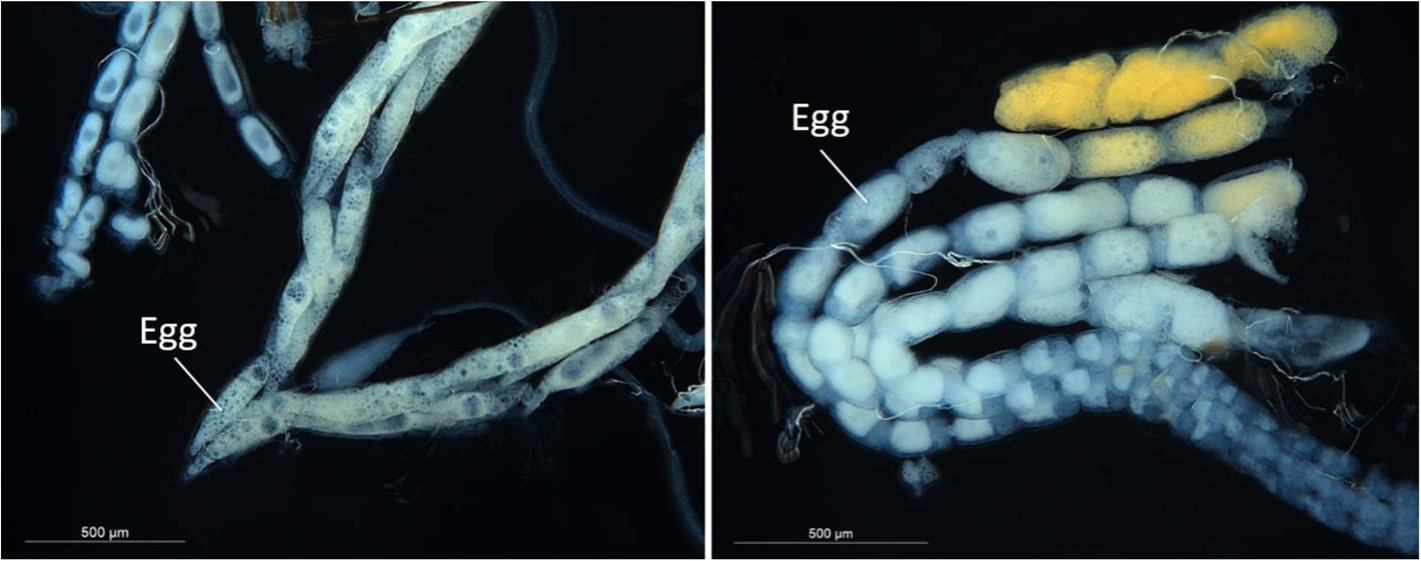
Ovaries and eggs of *Epicephala* sp. (**a**) (left) and *Epicephala* sp. (**b**) (right)

In females from species B, the seventh sternite was roughly trapezoid in shape and strongly wrinkled at the base (Fig. 4f). The antrum was broad and short, narrowest in the middle and approximately as long as the seventh sternite. The lamella postvaginalis was weakly bilobed with an enlarged sac before the ductus bursae, which was not sclerotized. Females from species A had a triangular seventh sternite. Both the seventh sternite and tergite were densely haired at the posterior end. The lamella postvaginalis was strongly bilobed and was often visible on the ventral surface of the abdomen (Fig. 4e). The antrum was long, as wide as the seventh tergite and asymmetrically more sclerotized on along the left lateral side which also featured a pronounced bulge above the ductus bursae and ductus seminalis. Unlike species B, in species A both the ductus bursae and ductus seminalis were strongly sclerotized. Differences were also seen in the shape of the females’ ovipositors. The apexes of the ovipositors in females from species B were shorter, blunter and roughly square tipped with a pronounced shoulder (Fig. 5d). By comparison females from species A had longer, narrower, “needle-like” ovipositors that curved backwards slightly towards the dorsal side (Fig. 5c). In addition, the opening from which eggs are extruded was on the dorsal side for species A and the ventral side for species B. The eggs and ovaries of both species also differed strongly in shape. The eggs of species A were “spindle” shaped; longer and thinner than the broader oblong shaped eggs of species B (Fig. 6).

### Pollination and oviposition observations

**Fig. 7:**
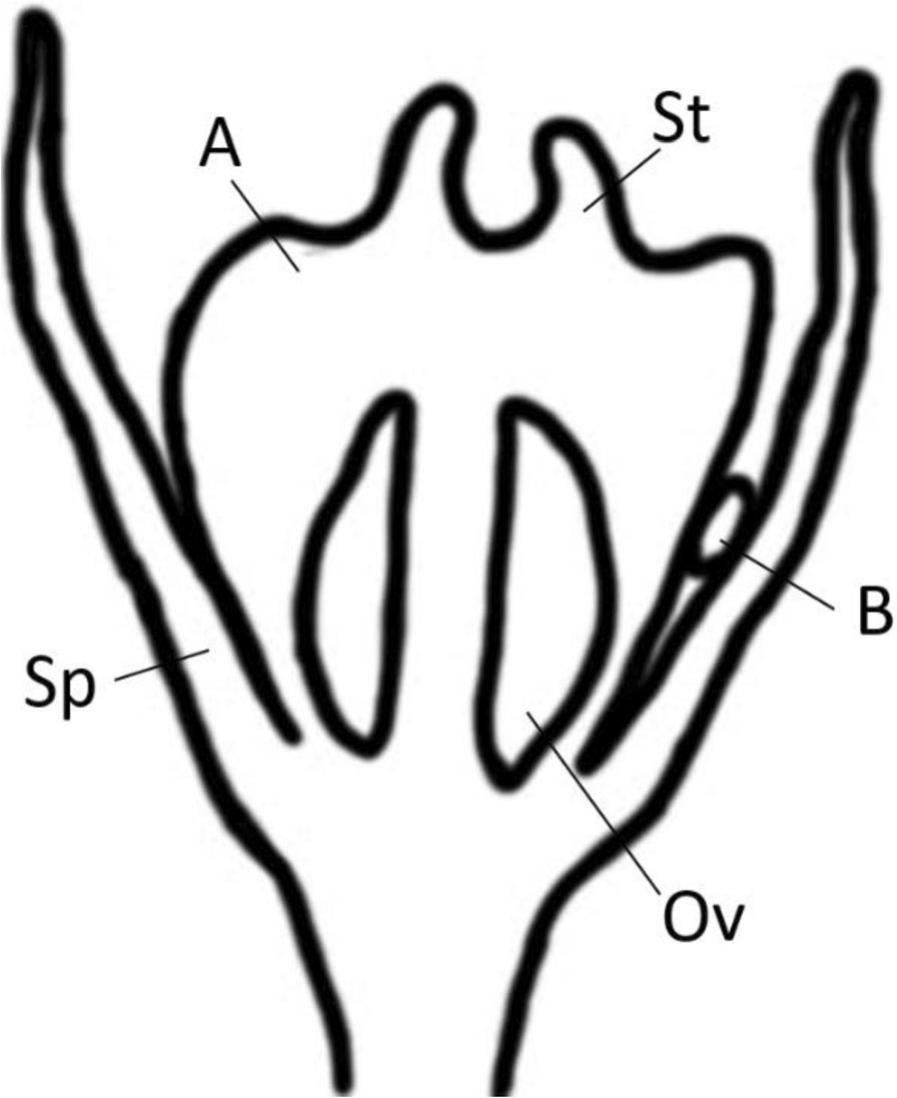
Cross sectional view of a female flower of *B. oblongifolia* showing the oviposition sites of *Epicephala* sp. A and B, ovules (Ov), sepals (Sp), and stigma (St)

In total we caught 77 female *Epicephala* moths visiting flowers between 13/09/17 and 22/04/18 at the Richmond site. Eighteen females were from species B and 59 were from species A. Females from both species were active over the same period of time and all captured females carried pollen on their proboscises (Fig. 5a-b). The mean number of pollen grains deposited by females from species B was 21 (*n* = 18) and 19 for species A (*n* = 59). There was no statistical difference between species A and B in the number of pollen grains deposited on flowers (*t* = − 0.69, df = 54, *p* = 0.49). Females from species A used their long needlelike ovipositors to deposit eggs into the tissue directly beneath the stigma and above the ovary (Fig. 7), resulting in a narrow scar in the plant tissue. In contrast, females from species B oviposited into the space between the ovary and the sepal and this did not result in any observable damage.

Although no *Epicephala* moths were active on the evening of 15/01/18 at the Shoal Bay site, we observed 10–20 *Herpystis* sp. ANIC1 ovipositing eggs onto the external surface of already developing *B. oblongifolia* fruits (< 5 mm). Captured *Herpystis* moths did not have pollen on their proboscises (*n* = 9) and were not seen to visit female or male flowers.

## Discussion

Taken together, our molecular and morphological data, along with behavioral observations, clearly indicate the presence of two species of *Epicephala* pollinator on *B. oblongifolia*. *Breynia* hosts these two closely related species in all six sampling sites, violating the traditional assumption of reciprocal specificity [79]. Both species carry pollen and were seen to actively pollinate female flowers and as such so there is no indication that either of the two is acting as a “cheat” within the mutualism. However, we also found that *Herpystis* sp. ANIC1 infests fruits, does not carry pollen and oviposits after fruit development has initiated, indicating that it is a non-pollinating seed parasite. *Breynia,* therefore, has two closely related co-existing pollinating moths and a third non-pollinating seed parasite species, from a distantly related moth lineage.

### Parasites

Seed parasites are not uncommon in the Phyllantheae [10, 46, 47] or in OPMs in general [80]. Moths in the genera *Peragrarchis* (Carposinidae), *Cryptoblabes* (Pyralidae) and *Tritopoterna* (Tortricidae) have been found in the fruits of *Glochidion* and have all been recorded to use multiple host plant species. *Herpystis,* the moth identified by our study, and *Tritopterna* both belong to the Eucosmini tribe of the Tortricidae [81]. However, they are not believed to be closely related [82] and our analysis of their COI genes supports this. Members of these two related genera seem to have independently adopted a seed-parasitic lifestyle on plants in the Phyllantheae.

*Herpystis* was sampled at 3/6 sites and was often as abundant as *Epicephala*. This raises the possibility that *Herpystis* may vary considerably in abundance across sites. Some *Breynia* populations may endure high seed predation by this species, whilst others escape the loss of reproductive effort. Selection could thereby differ between *Breynia* populations, creating a co-evolutionary mosaic [45]. Further sampling is required to determine the impact of parasitism on the mutualism and its evolutionary implications.

*Herpystis* species have been documented to infest the fruits of other *Breynia* sp. in Australia [82], but there are also records of them infesting the fruits of other unrelated plant taxa [83]. As such, it remains to be seen how widespread and host specific the associations between *Breynia* and *Herpystis* species are. In the OPMs occurring in *Yucca* spp., pollinators and closely related, co-occurring florivores have similar degrees of host specificity [49]. However, a study that compared the host specificity of *Epicephala* and non-pollinating seed parasitic moths in several species of *Glochidion* in Japan and Taiwan [46], found that *Epicephala* pollinators are more host specific than seed parasitic genera using the same host plant. The high specificity of *Epicephala* pollinators in *Glochidion* may be due to strong selection for host fidelity where multiple potential hosts occur [46]. High host fidelity may be driven by species specific floral traits that evolved to prevent heterospecifc pollen transfer between co-existing host plants. Such selection would not be imposed on non-pollinating seed parasites. To date, there has been only one study, in China, of host specificity in co-occurring *Breynia* species [34]. In that instance, pollinators were found to use multiple, although closely related and potentially hybridizing, host species. Therefore, it would be interesting to determine if the patterns of pollinator and parasite host specificity are consistent in co-occurring *Breynia* species, of which several occur in some tropical regions of Australia [84]. Such a study may help to further our understanding of the factors that promote species specificity in OPMs.

### Co-occurring pollinators

The two species identified here show obvious morphological differences in both their ovaries and in the male and female genitalia. The description and identification of *Epicephala* species relies heavily on genital morphology [54, 85, 86]. The large variation in genital morphology between species is common in *Epicephala* but unusual in the wider family Gracillariidae [54]. The reason for such marked variation between *Epicephala* species remains unclear, but could be related to species recognition [87], sperm competition [88, 89] or sexual conflict between males and females [90]. Whatever the cause, the morphology described here will prove useful for the future identification of these species. However, because of the lack of information on genital morphology in the described Australian *Epicephala* species, the identity of the two species will remain uncertain until a revision of the described Australian genera is completed.

The only previous study of *B. oblongifolia* pollination identified one *Epicephala* species from a single site in the Windsor Tablelands in Northern Queensland [14]. That study used genital morphology (data unpublished) to distinguish the number of species collected from each host plant species prior to sequencing [14]. Based upon this they identified and sequenced only a single *Epicephala* moth (species B) from *B. oblongifolia* [14]. Analysis of that sequence with our own clearly places the specimen collected from Northern Queensland within our species B. Species B comprises most of our samples, including ones collected from Southern New South Wales (Fig. 4). It is therefore likely that *Epicephala* species B has a latitudinal range of at least 2500 km along Australia’s east coast.

In New South Wales *Epicephala* species A and B co-occur at all our study sites over a distance of approximately 750 km. At three sites we found both species in fruits collected from the same individual plant (Table 1), suggesting that they are probably not using different host plant sub-populations. Additional sampling in Queensland will be required to determine if both species occur throughout the large geographic range of *Breynia oblongifolia*.

In some OPMs, particularly in *Ficus* spp*.*, multiple pollinators of a single host plant species can have distinct habitat or geographic distributions [24, 27, 35, 91]. In some instances, regional climatic variation may promote local adaptation and speciation in pollinators [35]. Geographic segregation in OPM pollinators on the same host can also result from allopatric speciation due to gene flow barriers [30, 33]. Several studies have sampled pollinators from *Ficus rubiginosa*, along the east coast of Australia, and the geographic range of this fig species is very similar to that of *Breynia oblongifolia* in coastal regions [27, 32, 35]. Those studies found a monophyletic group of five largely cryptic species of pollinator with a mixed pattern of geographic segregation and local co-existence. Studies on *F. rubiginosa* suggest that multiple factors including climate, historical biogeography and possible interactions with *Wolbachia* endosymbionts can influence pollinator diversity in OPMs. The distribution of the two pollinator species identified in our study was found to be entirely overlapping within our sampling area. Regional climatic adaptation therefore cannot explain their co-occurrence.

Our phylogenetic analysis and species delimitation models indicated that our species A is very closely related to the *Epicephala* sp. collected from *B. disticha* in New Caledonia [14] and that they may belong to the same species. However, as the two species delimitation models did not reach a consensus, we consider the question of whether these two *Epicephala* are in fact the same species or two closely related species remains a moot point. Addressing this requires further sampling and, in particular, detailed study of their genital morphology. Regardless, it seems likely that the co-occurrence of two *Epicephala* species on *B. oblongifolia* is consistent with a host switch. The ancestor of one of the two pollinators likely colonized *B. oblongifolia* from a related host, probably another *Breynia* species. Although it is possible that species A colonized *B. oblongifolia* from *B. disticha, or* vice versa, many alternative sequences of host shifts and speciation events are also imaginable. Ultimately, as we have data for only a few of the *Epicephala* species from the ~ 70 *Breynia* spp., it is not yet possible to determine the sequence of host shifts that resulted in these two pollinators sharing the same host.

Host switches are important factors in explaining the lack of strict sense co-speciation in OPMs [22, 26, 30]. They are well documented in *Epicephala-*Phyllantheae OPMs [30, 40] and, more generally, in the interactions between herbivorous insects and their plant hosts [41–44, 92]. The occurrence of two pollinator species on a single host in our own study is further evidence that plant-insect speciation within the *Epicephala-*Phyllantheae OPM is not entirely linked. In general, each major clade of the Phyllantheae that is involved in OPMs is host to a single unique clade of *Epicephala* [65], although there is at least one known exception [40]. Both *Epicephala* species identified by this study were nested within the *Epicephala* clade associated with *Breynia* spp. To date all *Epicephala* associated with *Breynia* species have been from this clade. As such, it is apparent that some degree of taxonomic matching is occurring in *Epicephala-*Phyllantheae OPMs [14, 40, 52] and is perhaps better defined as the co-divergence of species, or in this case genera and sub-genera [31].

### Competition between pollinators

Both *Epicephala* species identified by this study acted as pollinators and were present at all sites, although species A was less abundant than species B at 5/6 sites (Fig. 2). There are several examples of *Epicephala* species co-existing locally on the same *Glochidion* host plants [30, 62], but *Breynia* has received less study [34]. Some evidence suggests that co-occurring species of pollinating *Epicephala* may be in competition [34]. At least one species of *Epicephala* seems to have displaced another ancestral species following a host shift [40]. As such, in some instances, competition between pollinators may result in the extinction of a less competitive species.

In OPMs, adult pollinators may compete for access to limited and ephemeral oviposition sites in the form of female flowers. In this situation competition is most likely pre-emptive, meaning that successful colonizers, in this case eggs and larvae, cannot be displaced from a flower, even by a superior competitor. Competition between pollinators in OPMs may therefore most closely resemble a lottery model, in which female flowers are limited, larvae cannot be displaced from those flowers and colonization of a flower by a larva is on a first-come, first-served basis [93, 94].

Lottery models alone, however, cannot explain co-existence as under these circumstances alone the species with highest population growth rate will eventually drive the other to extinction [95]. Co-existence under lottery-type competition can only occur if there is sufficient environmental heterogeneity to allow each species to have the highest population growth rates at a particular time or place in their shared habitat [96]. For example, closely related species of stingless bee co-exist on floral resources through specializing on high and low host plant densities [97] and similar patterns have been found in ants [98]. As such, competition between co-pollinators may be limited by subtle differences in their ecology and niche space [99].

The two *Epicephala* species in our study had distinctly shaped ovipositors and oviposition sites. Species A uses it’s longer “needle-like” ovipositor to deposit eggs into the plant tissue near the flower ovules. The eggs of species A are also long and thin in shape and this is most likely an adaptation to allow eggs to pass through the narrow ovipositor. Conversely, species B inserts its eggs between the sepal and the ovule wall using a shorter blunter ovipositor. The eggs of species B are correspondingly wider. Our finding of co-occurring pollinators with contrasting oviposition strategies is paralleled in other *Breynia* species [34], suggesting that this trait may be important for pollinator co-existence in *Breynia*.

Previous studies have suggested that ovipositing into the plant tissue may reduce egg and larval mortality, potentially giving internally ovipositing species a competitive advantage [34]. The internally ovipositing species A was the species most commonly collected during our pollination observations. However, the externally ovipositing species B was the species that emerged most frequently from fruits at the majority of study sites (Fig. 2). Ovipositing into plant tissue may in fact result in greater floral damage, a higher incidence of floral abscission and increased larval mortality, as seen in some *Yucca* and Phyllantheae [100–102]. Under these circumstances, externally ovipositing females might gain a competitive advantage. Whilst certain oviposition strategies give some species greater growth rates than their competitors, this does not explain how two species can co-exist, as seen in this study and others [34]. We hypothesize that the two oviposition modes seen here may result in different larval mortality depending on their environmental context. Such differences may allow for sufficient heterogeneity in growth rates to explain the co-existence of both species in line with the competition versus colonization theory of species co-existence [98]. It may also help to explain the variability in relative abundance of species across sites (Fig. 2). Further work is therefore required to determine the source of environmental heterogeneity that allows these two species, with very similar ecological niches, to co-exist over such a large area.

## Conclusions

The results of our investigation add to the growing understanding of *Epicephala* diversity in OPMs. Fewer *Epicephala* species have been described to date than are estimated to exist [54]. Of the Phyllantheae plant species involved in OPMs that have been investigated, several are associated with multiple *Epicephala* pollinators as well as numerous parasitic insects. *Epicephala* diversity may therefore number many hundreds of species, representing a truly outstanding example of plant-insect co-divergence.

The finding of multiple species of highly adapted pollinators co-occurring on the same host is in keeping with a more nuanced view of co-evolutionary processes in OPMs and plant-insect interactions in general. Closely interacting species can show high levels of reciprocal adaptation [31, 103, 104] and varying degrees of strict co-speciation [20, 21] but are highly dynamic, frequently being affected by complex ecological and biogeographic factors such as host shifts [26, 27, 30, 105, 106]. These factors can create incongruences between plant-insect phylogenies but are themselves important processes in the diversification and evolution of species.

## Additional file

Additional file 1: Neighbor-joining consensus tree of 135 *Epicephala* COI sequence subunits aligned to the only other *Epicephala* species previously sampled from *Breynia oblongifolia; Epicephala* sp. ex. *Breynia oblongifolia* (NCBI: FJ235381.1) [14]. Branch labels show percentage consensus support after 200 bootstrap replicates. (PDF 218 kb)

## Abbreviations

GMYC: Generalized Mixed Yule Coalescent models
NJ: Neighbor Joining
OPM: Obligate pollination mutualism
PTP: Poisson Tree Processes
