# Supplementary figures and images for: Two’s company, three’s a crowd: co-occurring pollinators and parasite species in Breynia oblongifolia (Phyllanthaceae)
Source: BMC Evol Biol. 2018 Dec 14;18:193. doi: 10.1186/s12862-018-1314-y (PMC6295073; doi:10.1186/s12862-018-1314-y)

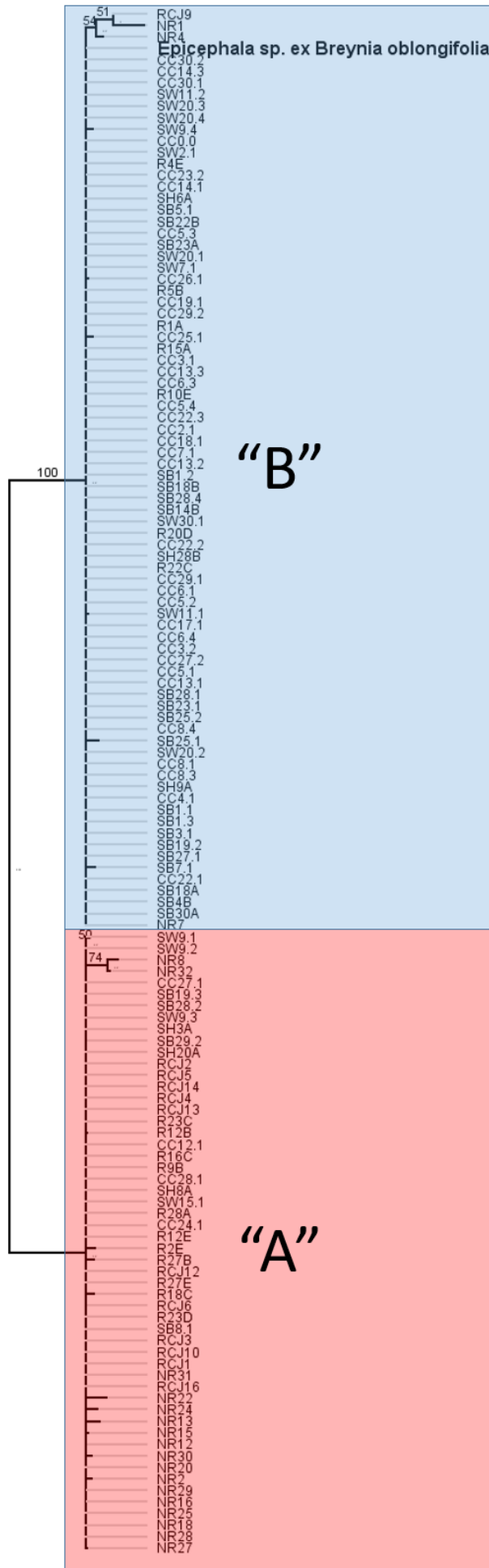

Supplement: Supplementary file 1 — Neighbor-joining consensus tree of 135 Epicephala COI sequence subunits aligned to the only other Epicephala species previously sampled from Breynia oblongifolia; Epicephala sp. ex. Breynia oblongifolia (NCBI: FJ235381.1) [14]. Branch labels show percentage consensus support after 200 bootstrap replicates. (PDF 218 kb) [file 12862_2018_1314_MOESM1_ESM.pdf]
